# Cross-Continuum Tool Is Associated with Reduced Utilization and Cost for Frequent High-Need Users

**DOI:** 10.5811/westjem.2016.11.31916

**Published:** 2016-12-09

**Authors:** Lauran Hardin, Adam Kilian, Leslie Muller, Kevin Callison, Michael Olgren

**Affiliations:** * Trinity Health-Michigan dba Mercy Health Saint Mary’s, Grand Rapids, Michigan; † University of Utah Health Care, Department of Internal Medicine, Salt Lake City, Utah; ‡ Grand Valley State University, Economics Department, Grand Rapids, Michigan; § National Center for Complex Health and Social Needs, Camden, New Jersey

## Abstract

**Introduction:**

High-need, high-cost (HNHC) patients can over-use acute care services, a pattern of behavior associated with many poor outcomes that disproportionately contributes to increased U.S. healthcare cost. Our objective was to reduce healthcare cost and improve outcomes by optimizing the system of care. We targeted HNHC patients and identified root causes of frequent healthcare utilization. We developed a cross-continuum intervention process and a succinct tool called a Complex Care Map (CCM)© that addresses fragmentation in the system and links providers to a comprehensive individualized analysis of the patient story and causes for frequent access to health services.

**Methods:**

Using a pre-/post-test design in which each subject served as his/her own historical control, this quality improvement project focused on determining if the interdisciplinary intervention called CCM© had an impact on healthcare utilization and costs for HNHC patients. We conducted the analysis between November 2012 and December 2015 at Mercy Health Saint Mary’s, a Midwestern urban hospital with greater than 80,000 annual emergency department (ED) visits. All referred patients with three or more hospital visits (ED or inpatient [IP]) in the 12 months prior to initiation of a CCM© (n=339) were included in the study. Individualized CCMs© were created and made available in the electronic medical record (EMR) to all healthcare providers. We compared utilization, cost, social, and healthcare access variables from the EMR and cost-accounting system for 12 months before and after CCMs© implementation. We used both descriptive and limited inferential statistics.

**Results:**

ED mean visits decreased 43% (p<0.001), inpatient mean admissions decreased 44% (p<0.001), outpatient mean visits decreased 17% (p<0.001), computed tomography mean scans decreased 62% (p<0.001), and OBS/IP length of stay mean days decreased 41% (p<0.001). Gross charges decreased 45% (p<0.001), direct expenses decreased 47% (p<0.001), contribution margin improved by 11% (p=0.002), and operating margin improved by 73% (p<0.001). Patients with housing increased 14% (p<0.001), those with primary care increased 15% (p<0.001), and those with insurance increased 16% (p<0.001).

**Conclusion:**

Individualized CCMs© for a select group of patients are associated with decreased healthcare system overutilization and cost of care.

## INTRODUCTION

### Healthcare Overutilization is a Costly Problem

As the United States grapples with steeply rising healthcare cost, payers, providers, and policymakers seek to improve the efficiency of healthcare delivery.^1^ We are challenged by the problem of costly healthcare overutilization by high-need, high-cost (HNHC) patients – those requiring complex and multifaceted care with frequent access to the healthcare system.^1^ Although these patients represent a relatively small proportion of the population, their care is associated with disproportionately high expenditures. For example, the top 1% of patients accounts for more than a fifth of all healthcare spending, and the top 5% accounts for nearly half.^2^,^3^ Effective intervention in this population has the potential to reduce waste and improve millions of lives.^4^ We tested the development and use of CCMs© to reduce overutilization in high-need patients.

### High-Need, High-Cost Patients

The complexity of HNHC patients often extends beyond medical diagnoses to include community, behavioral, cultural, addiction, and socioeconomic challenges.^1^,^2^ Compared to the general population, these patients have a higher prevalence of chronic physical and psychiatric illnesses that require both immediate interventions and long-term care, present with complaints more appropriate for primary care, have higher rates of hospitalization and mortality, are ethnically diverse, have varied health and personal histories, and are more likely to have enduring problems such as poverty, homelessness, hunger, loneliness, illiteracy, lack of transportation, limited mental capacity, legal problems, and substance addiction.^4^–^6^ Studies suggest that the complexity of these patients’ medical and/or socioeconomic maladies hinders their ability to navigate the healthcare system, contributing to the cycle of overutilization.^7^ Fragmentation in the healthcare system also drives overutilization. The increasing number and complexity of visits in a healthcare system that are not organized around meeting the multifaceted physical, behavioral, and social needs of these high-need individuals results in fragmented and episodic care.^4^,^8^ Patients cycle through multiple institutions (such as emergency departments [ED], inpatient [IP] units, outpatient clinics, detox centers, homeless shelters, and jails) that are often disconnected from one another, leading to an expensive, inefficient healthcare environment that fails these patients.^4^,^9^

### Seeking a Solution to Overutilization

A “best practice” approach has proven elusive, with the majority of care remaining fragmented, uncoordinated, and reactive.^2^ Interventions to improve management and reduce utilization have largely focused on adding care managers to directly work with the patient to enhance access and care coordination. Approaches have included individualized care plans and intensive case management,^10^–^22^ healthcare education, improving access to primary care,^23^–^25^ patient home follow up,^26^,^27^ triaging patients and routing non-urgent cases to alternative services, and managed care-level interventions.^28^ Several models, such as the Commonwealth Care Alliance, CareMore, CareOregon, the Everett Clinic, and Marshfield Clinic, have adapted a range of approaches that include medical homes in safety-net clinics, multidisciplinary case management, patient stratification to better target care delivery, early intervention strategies, and vigorous discharge follow up.^4^ Although many programs have improved quality or reduced care utilization, their impacts on costs have been inconsistent.^31^,^32^

A growing need remains for initiatives with an innovative model that improves care delivery and beneficiary experience, while reducing unnecessary spending for all patients, especially for this vulnerable population with complex medical and social needs.^4^ The lack of a consistent understanding of the characteristics of this heterogeneous high-need population, which underlying issues drive high-utilization behavior and which subgroups offer the greatest opportunity for impact, all hamper efforts to innovate and implement effective interventions that improve healthcare delivery.^2^,^4^ Much remains unknown about how HNHC patients interact with the healthcare system, what services they receive, and what outcomes result.^4^ If we can understand more about the care they need and what is working, we can design more targeted, coordinated, and effective clinical services.^4^

### Our Approach and Goals

Whereas most interventions focus on *changing the patient,* our approach to improve the effectiveness, efficiency, and value in care was to focus on innovating a replicable intervention that *changes the system of care around these patients* to effectively identify and target the true root causes driving the high-utilization behavior.

The CCM© is a cross-continuum succinct tool that addresses fragmentation in the system by linking providers to a consistent cohesive individualized analysis of a patient’s root causes for frequent use of costly acute health services. The CCM© is linked to a pop-up alert that fires the first time a provider opens the medical record. It is a guide that demystifies the complexity of a frequent user’s clinical presentation and utilization pattern. The provider is thus equipped with a comprehensive analysis of underlying root causes contributing to return visits with supporting data. The CCM© allows each provider to examine the history and considerations for care from the patient’s cross-continuum of healthcare providers, so that he/she can be better informed regarding how to provide the most appropriate and consistent care for patients with complex issues. The CCM© captures the patient’s longitudinal story and brings forward considerations to improve delivery of care.

In this article, we describe 1) a system-focused, root cause-based intervention, 2) our process for creating and implementing CCMs©, 3) the profile of our patient population, and 4) utilization, financial, social, and healthcare access outcome measures after the CCM© was administered. Our aim for sharing our approach is to advance understanding of the heterogeneous HNHC patient population.

## CREATION OF THE COMPLEX CARE MAP© TOOL

**Figure 1:**
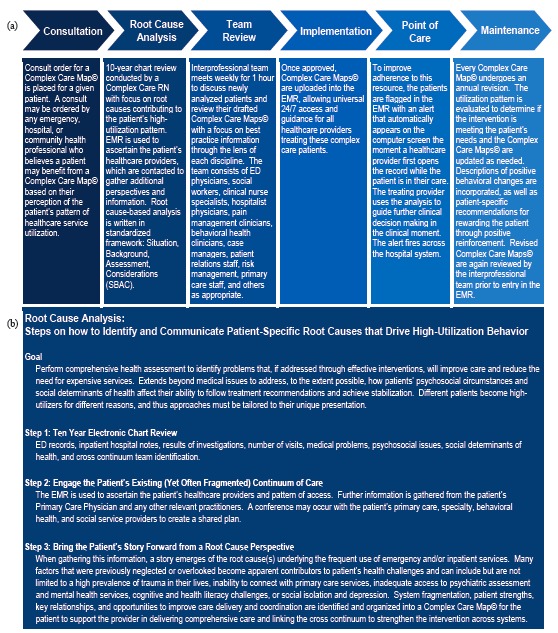
Creating and implementing Complex Care Maps©: (a) General overview (b) process for conducting root cause analysis of drivers that underlie high-utilization behavior.

**Figure 2:**
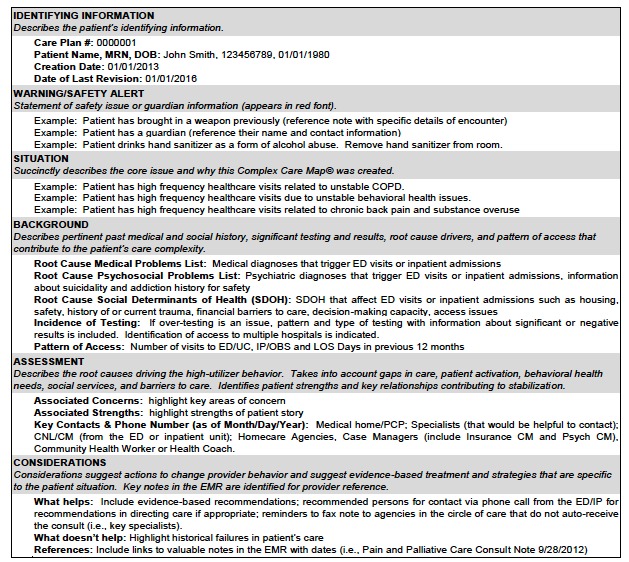
Complex Care Map© architecture.

**Figure 3:**
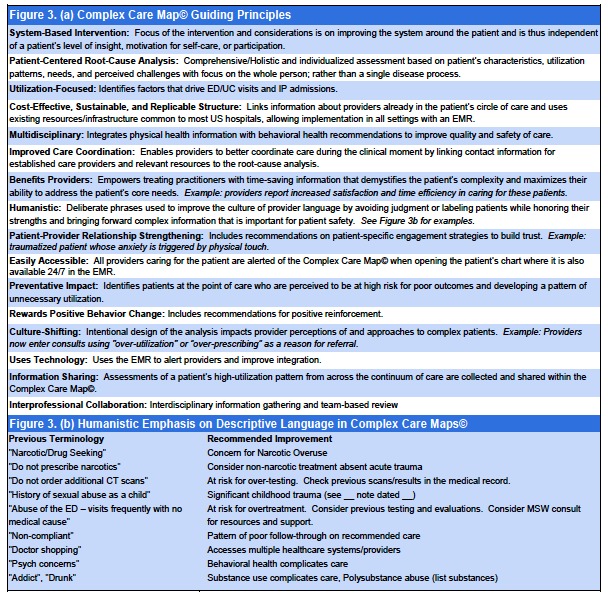
Distinguishing features: (a) Guiding principles. The Complex Care Map© incorporates several important and distinguishing features, some of which are known to be shared by high-performing approaches; (b) humanistic emphasis on descriptive language in Complex Care Maps©.

A master’s prepared clinical nurse leader (CNL) created a Complex Care Resource Center where, under her leadership, tools were developed to complete a record review, uncover root causes of instability, capture the cross-continuum team, and identify key drivers that may improve outcomes for the patient. The CNL and ED medical director co-led an interprofessional Complex Care Committee to develop and maintain the CCMs© (Figures 1, 2, and 3).

## METHODS

### Context

Our analysis was designed to explore if a CCM© would improve quality of healthcare delivery, reduce inappropriate overutilization of costly acute care services, and improve social and healthcare access and patient outcomes. A Complex Care nurse chaired the intervention and oversaw the interdisciplinary team.

### Study of the Intervention

#### Study Design

The intervention was designed as a quality improvement project that followed high health system users for 12 months pre- and post-intervention where each subject served as his/her own historical control. We used retrospective data for comparison. This project was deemed as a Clinical Quality Improvement Initiative by the Mercy Health Institutional Review Board (IRB) and as such was not formally supervised by the IRB per their policies.

#### Setting

The analysis was conducted between November 2012 and December 2015 at Mercy Health Saint Mary’s in Grand Rapids, Michigan, an inner city tertiary care hospital with greater than 80,000 annual ED visits. Because of its location, a large number of patients are homeless, unemployed or receiving social assistance, have complex and/or chronic medical, psychiatric, and substance use problems.

#### Subject Population

Any patient referred to have a CCM© was enrolled in the project. Referrals could be made by any hospital, emergency, or community health professional who believed a patient could benefit from a Complex Care Map© based on their perception of the patient’s pattern of healthcare service utilization. Additional inclusion criteria included three or more visits to the hospital within the prior 12 months and age of 18 years or older. There were no exclusion criteria. Subjects were withdrawn from the analysis prior to completion if they died or were known to have moved away within 12 months after initiation of a CCM©. In total, 355 cases were enrolled, and 16 cases were withdrawn due to death prior to 12 months after implementation of the intervention.

### Measures

#### Outcome Variables

The present analysis investigated whether implementing CCMs© could reduce healthcare service utilization and costs (primary objectives) and improve social and healthcare access issues (secondary objectives).

#### Primary Outcome Variables

Our analysis had two sets of primary outcome measures. One set focused on Healthcare Service Utilization: Emergency Department/Urgent Care (ED/UC) Visits, Observation/Inpatient (OBS/IP) Admissions, OBS/IP Length of Stay (LOS), Computed Tomography scans Ordered. We obtained healthcare service utilization data from the hospital’s inpatient and outpatient utilization databases and cost accounting system. The other set focused on healthcare service costs: Gross charges and expenses, ED service charges and expenses, IP service charges and expenses, outpatient service charges and expenses. Healthcare cost data were retrieved from the cost accounting system.

#### Secondary Outcome Variables

Our study had one set of secondary outcome measures. These measures focused on social and healthcare access issues: lacks safe housing, lacks medical insurance, lacks primary care. We obtained social and healthcare issues data from extensive review of the patient’s EMR and reports of collateral contacts/patient’s healthcare providers. “Lacks safe housing” was defined as living on the streets, in shelters, or in an abandoned building for the majority of the time.

#### Descriptive Variables

We describe a comprehensive set of baseline characteristics for the high-frequency complex patient population in our analysis grouped into several categories (Table 1): demographic, social, healthcare access, mental illness, and healthcare utilization variables. History of trauma was defined as history of a severely distressing event that caused overwhelming stress or psychological trauma such as, although not limited to, physical or sexual assault, serious bodily harm, natural disasters, or witnessing fatalities. Baseline patient characteristics were obtained from extensive review of each patient’s EMR.

### Analysis

#### Statistical Procedures

All data were extracted from the hospital’s EMR system, compiled in a Microsoft Excel spreadsheet, and then stored as de-identified data in REDCap prior to being transported to Stata version 14SE (STATA Corp). As this was a paired sample study with data collected on the same patients (before and after CCM© implementation), we used Wilcoxon signed rank tests and McNemar’s chi-square test to examine whether the difference in pre- and post-outcome measures were statistically significant. McNemar’s chi-square test is used for binary variables and the Wilcoxon test is used for count data. Tests were two-sided and a p value < 0.05 was considered statistically significant. In an effort to examine distributional differences in utilization changes from the pre- to post-period, we conducted an analysis using patients in the 25^th^ and 75^th^ percentiles of the distribution for each utilization outcome.

Examining costs from the hospital’s perspective is an essential step because it is unlikely that any hospital would implement a new program that was not cost effective at the health system level.^30^ We report financial data (rounded to the nearest dollar) and acknowledge that hospital charges, billing, and revenue figures may vary widely among hospitals because of unique combinations of patient mix, payer mix, and institutional mission, although it is the changes in these variables that we emphasize.

## RESULTS

### Baseline Patient Characteristics

Table 1 reports characteristics of the sample. In many respects, patients were typical of most high-utilizer groups: a large proportion had Medicaid (42%), were dual-eligible Medicare/Medicaid (17%), or were uninsured (17%). A high percentage of patients also had history of mental health diagnoses, including suicidality (40%), trauma (48%), substance use disorder (66%), and/or psychiatric diagnosis (75%). Eighteen percent of high-utilizers were homeless.

Patients in this study also had characteristics that differ from most other studies. The mean age was 40 years (not shown in Table 1), with 72% of patients less than 50 years old. Furthermore, 35% were from healthcare systems outside of Mercy Health (home-based primary care provider (PCP), other PCP, and one-third of the Resident Clinic), while 46% were from PCPs inside the Mercy system. Twenty-five percent of patients had three or more years of prior frequency. In our work with complex patients, we have found the characteristics of those with multiple years of frequency require a comprehensive approach for stabilization; except for Johnson et al. (2015), who included data on frequency for one year prior to intervention, we are not aware of any other studies that consider past utilization.^34^

#### Patient Outcomes of Intervention

Table 2a reports the difference in both primary and secondary outcomes pre- and post-implementation of the CCM©. The primary outcomes include measures of healthcare utilization and healthcare costs. Using data from cost-accounting classifications, total visits decreased by 37%, with ED visits decreasing 43%, IP visits decreasing 44%, and OP visits decreasing 17%. Using data from the quality improvement database, ED/UC visits decreased 30% and IP/OBS utilization decreased 49%. The number of CTs decreased 62% and LOS decreased 40.5%. All p-values for healthcare utilization outcomes were <0.001. Gross charges decreased 45%, ED charges decreased 48%, IP charges decreased 43%, and OP charges decreased 47% (p<0.001). Total direct expenses decreased 47%, as did expenses for ED (50%, p<0.001), IP (45%, p<0.001), and OP (50%, p<0.001). The total contribution margin increased 11% (p<0.001), with the ED contribution margin increasing 76% (p<0.001) and the OP contribution margin increasing 86% (p<0.001). The total operating margin increased 73%, with the ED operating margin increasing 58% (p<0.001) and the OP operating margin increasing 60% (p<0.001). The differences between the pre- and post-intervention IP contribution margin and operating margin were statistically insignificant.

The secondary outcomes include social and healthcare access variables. Differences for all secondary outcomes were statistically significant, with a p-value <0.001. After intervention, the number of patients with housing increased 14%, patients with an identifiable PCP increased 15%, and patients with insurance increased 16%.

#### Distributional Analysis of Patient Outcomes

To examine the extent to which our results may be driven by regression to the mean and not to the intervention itself, we divided our sample into quartiles based on each outcome and repeated the pre- to post-period analyses reported in Table 2a. For this distributional analysis, we chose to focus on utilization outcomes, as those would provide the clearest evidence of the influence of natural variation in our findings. Results are presented in Table 2b and, while not definitive, do provide evidence that regression to the mean in our sample is minimal. The first four columns display pre- and post-intervention mean utilization rates for the lowest utilizers in the sample, while the last four columns include the same information for the highest utilizers. Unsurprisingly, the highest utilizers experience the largest post-intervention reductions in the utilization outcomes, many on the order of 50%, while the lowest utilizers appear to be largely unaffected by the intervention. Importantly, we see little indication of movement towards the mean for the lowest utilizers in the post-period, lending support to the effectiveness of the CCM©. Four of the outcomes for the lowest utilizers show no statistically significant change from the pre- to post-period, and the remaining changes – while statistically different from zero – are small in magnitude.

## DISCUSSION

### Summary

We implemented an interprofessional, replicable, cost-effective process to intervene with HNHC patients. In this article, we share information about the people with the most significant healthcare needs and the services they use. We describe an EMR-based care delivery intervention that is associated with lower-than-average costs. We improved social and healthcare access outcomes by changing the system around complex patients.

### Interpretation of Key Findings

#### Intervention

This paper describes a successful approach to stabilize HNHC patients. The CCM© is unique in that it combines the power of the patient story with interprofessional input and focuses on cross-system collaboration to improve outcomes. This intervention, which was associated with a 72.5% increase in operating margin, may prove particularly valuable as health systems shift further into risk-based contracts. Rather than creating another care management and cost infrastructure, the intervention is primarily managed by existing resources in the healthcare system and operates by improving efficiency through coordination of existing providers. Cost for implementation is minimal because it uses existing technology available to most healthcare systems. Keeping referral criteria open allows providers to identify at-risk patients at the early stages of high-utilization behavior, hopefully preventing patients from becoming consistent frequent users. We believe this is significant because the greatest long-term cost savings will come from reducing the development of new high-utilizer patients. Although not quantified in this analysis, providers report a high level of satisfaction with having an easy process to identify at-risk patients in the moment of interaction without having burdensome documentation to complete to generate intervention.

In addition to reductions in utilization, patients in the study experienced reductions in length of stay and in the number of CTs performed. Providers report that CCMs© save time and help them link with the cross-continuum team already caring for the patient. Efficiency in delivery may contribute to reduction in length of stay but this was not explored in the analysis. Quantity of CTs and previous results are specifically included in the CCMs© because a pattern of frequent investigations was noted in the population. Providers now make referrals of patients specifically due to noted “over-testing.” Additional investigation into the drivers of these changes in practice warrant future study.

#### Description of the Sample

Predominant characteristics of our sample include prevalence of fragmentation between cross-continuum providers and prevalence of mental illness, substance use, and trauma. Individuals in this population were typically younger than expected with 72% being less than 50 years old. Annual analysis of high-frequency patients (10 or greater ED visits OR four or greater inpatient admissions) at Mercy Health has shown that 70% of the population is less than 60 years old.^35^ Surprisingly, we did not find a prevalence of medical disease driving high-frequency access in this population. We observed an important trend of a portion of this population using multiple healthcare systems; as health systems move further into risk-based contracts, it is important to consider the movement of patients between systems.

### Next Steps

After development and successful implementation with high levels of engagement at our institution, a toolkit was developed to translate implementation knowledge, and standard evidence-based CCMs© were created for common subpopulations. CCMs© are currently being piloted at 26 Trinity Health hospitals across six different states in a web-based learning collaborative.^33^

## LIMITATIONS

Our quality improvement analysis compares pre-intervention and post-intervention data whereby all patients were used as their own control. Observational design has potential for confounders and we do not report risk-adjusted data. Some of the effects could be attributed to a natural reduction in healthcare utilization and costs over time (i.e., regression to the mean). To address this limitation, we 1) performed a distributional analysis of the utilization outcomes, which provides evidence that regression to the mean in our sample is minimal; and 2) included the number of years subjects were high-utilizers prior to intervention. Additionally, referral through a consultation process may introduce bias into the sample; however, we do not consider this a weakness but rather a strength of the intervention since it places value on a professional’s assessment of a patient’s level of complexity within the clinical moment, which we believe is a valuable way to identify patients whose complex needs are not being met. Our analysis is restricted to a single healthcare system, which reduces generalizability of the results to other settings, especially considering that frequent users could use more than one hospital network for access. Despite these limitations, we believe the main findings of our analysis provide important contributions for improving the efficiency of healthcare delivery to HNHC patients.

## CONCLUSION

CCMs© for a select group of patients were associated with decreased healthcare system overutilization and cost of care.

## Figures and Tables

**Table 1:** Baseline characteristics (n = 339) for the high-frequency complex patient population.

	% (No.)
Demographic variables	
Age group	
18–29	17.40 (59)
30–39	31.56 (107)
40–49	23.60 (80)
50–59	18.58 (63)
60–69	6.49 (22)
70–79	2.06 (7)
80+	0.29 (1)
Gender	
Male	59.29 (201)
Female	40.71 (138)
Race	
White	63.72 (216)
Black or African American	30.09 (102)
Hispanic or Latino	5.01 (17)
Asian	0.00 (0)
American Indian or Alaska Native	1.18 (4)
Native Hawaiian or Pacific Islander	0.00 (0)
More than 1 race	0.00 (0)
Social variables	
Housing*	
Yes	81.42 (276)
Housed	73.45 (249)
AFC/AL	3.24 (11)
Long-term care	0.59 (2)
With family & friends	2.95 (10)
Transient hotel	1.18 (4)
None	18.58 (63)
Crisis house	0.29 (1)
Homeless	18.29 (62)
Healthcare access variables	
Identifiable PCP*	
Yes	81.12 (275)
MHPCMH	22.71 (77)
Resident clinic	8.26 (28)
Community benefit clinic	17.11 (58)
Home based PCP	0.88 (3)
Long term care PCP	0.59 (2)
Other PCP	31.56 (107)
None	18.88 (64)
Insurance Type*	
Insured	83.48 (283)
Private/commercial	12.09 (41)
Medicare	12.09 (41)
Medicaid	42.18 (143)
Dual-eligible (Medicare/Medicaid)	17.11 (58)
Uninsured	16.52 (56)
Healthcare utilization variables	
Years of prior frequency	
1	35.99 (122)
1–2	21.83 (74)
2–3	17.40 (59)
>3	24.78 (84)
Type of frequency	
ED	43.95 (149)
Inpatient	2.36 (8)
Both	53.69 (182)
Mental illness variables	
Hx of suicidality (yes)	40.1 (136)
Hx of trauma (yes)	48.1 (163)
Hx of substance use disorder (yes)	66.1 (224)
Hx of any psychiatric diagnosis (yes)	74.6 (253)

*Hx,* history; *AFC/AL,* adult foster care or assisted living; *MHPCMH*, Mercy Health patient-centered medical home; *ED*, emergency department; *PCP*, primary care physician.

* Designates Variable with 12-mo After Comparison.

**Table 2:** (a) Patient outcomes of intervention (n=339) pre- and post-implementation of the Complex Care Map©.

		Means				
		
Outcomes		Pre	Post	Difference	% Change	p-value
Healthcare utilization		Means (no.)				
Visits						
Total		14.903	9.322	−5.581	−37.4	<0.001
ED		10.245	5.862	−4.419	−43.1	<0.001
IP		1.295	0.720	−0.575	−44.4	<0.001
OP		3.362	2.780	−0.582	−17.3	<0.001
Total		11.826	7.997	−3.829	−32.4	<0.001
ED/UC		10.319	7.233	−3.086	−29.9	<0.001
OBS/IP		1.507	0.764	−0.743	−49.3	<0.001
CT scans						
Total		1.481	0.563	−0.918	−62.0	<0.001
		Means (days)				
LOS						
OBS/IP		5.850	3.481	−2.369	−40.5	<0.001
Healthcare costs		Means ($)				
Gross charges						
Total		39,254	21,491	−17.764	−45.3	<0.001
ED		13,121	6,831	−6,290	−47.9	<0.001
IP		20,768	11,795	−8,973	−43.2	<0.001
OP		5,365	2,864	−2,501	−46.6	<0.001
Direct expenses						
Total		10,956	5,788	−5,168	−47.2	<0.001
ED		3,009	1,492	−1,517	−50.4	<0.001
IP		6,556	3,597	−2,959	−45.1	<0.001
OP		1,390	699	−691	−49.7	<0.001
Contribution margin						
Total		1,134	1,253	119	10.5	0.002
ED		−770	−182	589	76.4	<0.001
IP		2,172	1,472	−700	−32.2	0.338
OP		−268	−37	231	86.0	0.004
Operating margin						
Total		−2,573	−707	1,866	72.5	<0.001
ED		−2,244	−948	1,296	57.7	<0.001
IP		475	562	87	18.3	0.771
OP		−803	−321	482	60.0	<0.001
Social variables						
Housing	Yes	81.4	92.9	11.5	14.1	<0.001
Healthcare access						
Identifiable PCP	Yes	81.1	93.2	12.1	14.9	<0.001
Insurance type	Insured	83.5	96.5	13.0	15.6	<0.001

Pre (12-mo before); Post (12-mo after).

*ED,* emergency department; *IP,* inpatient; *LOS,* length of stay; *OP,* Outpatient, Observation Admissions and Urgent Care Visits and Outpatient Radiology.

**Table 2:** (b) Distributional analysis of patient outcomes.

	25th Percentile				75th Percentile			
		
	Pre	Post	Difference	p-value	Pre	Post	Difference	p-value

Healthcare utilization	Values (no.)				Values (no.)			
Visits								
Total	5.621	5.770	0.149	0.362	27.341	15.841	−11.500	<0.001
ED	3.000	3.443	0.443	0.736	20.000	10.067	−9.933	<0.001
IP	0.000	0.231	0.231	<0.001	3.538	1.528	−2.010	<0.001
OP	0.000	0.588	0.588	<0.001	9.330	6.247	−3.083	<0.001
Total	5.764	7.180	1.416	0.462	27.678	17.411	−10.267	<0.001
ED/UC	3.112	4.051	0.939	0.217	20.122	11.800	−8.322	<0.001
OBS/IP	0.000	0.150	0.150	<0.001	3.642	1.545	−2.097	<0.001
CT scans								
Total	0.000	0.341	0.341	<0.001	3.212	0.826	−2.386	<0.001
LOS	Values (days)				Values (days)			
OBS/IP	0.000	0.571	0.571	<0.001	17.772	9.609	−8.163	<0.001

Pre (12-mo before); Post (12-mo after).

*ED,* emergency department; *IP,* inpatient; *LOS,* length of stay; *OP,* Outpatient, Observation Admissions and Urgent Care Visits and Outpatient Radiology.
